# Nanotherapeutic smart approaches for combating Alzheimer’s disease and overcoming existing obstacles: A novel eco-friendly green approach

**DOI:** 10.1016/j.toxrep.2025.101906

**Published:** 2025-01-27

**Authors:** Ahmed M. Almehdi, Doha H. Aboubaker, Rania Hamdy, Ali El-Keblawy

**Affiliations:** a Department of Applied Biology, College of Sciences, University of Sharjah, P.O.Box 27272, Sharjah, UAE; b Research Institute for Sciences and Engineering (RISE), University of Sharjah, P.O. Box 27272, Sharjah, UAE; c Medicinal and Aromatic Plants Dept., Pharmaceutical and Drug Industries institute, National Research Centre, Cairo, Egypt; d Department of Biology, Faculty of Science, Al-Arish University, Egypt

**Keywords:** Biogenic approach, Green chemistry, Metallic nanoparticles, Alzheimer's disease

## Abstract

The scientific community has united to raise awareness of Alzheimer's disease (AD) as a critical condition for future generations because recent predictions indicate that it will become common among the elderly within a few years. Nevertheless, the intricacies of the disease's progression demand exhaustive investigations to unravel its potential mechanisms. Only then can clinicians develop more efficacious therapeutic strategies. Cognitive impairment caused by amyloid aggregation, the development of hyperphosphorylated neurofibrillary tangles, and a malfunctioning cholinergic system are the hallmarks of AD. Even after the disease has started, brain tissue integrity may degenerate. The physiological characteristics of the highly selective blood-brain barrier and the electrostatic charge of the nanoporous extracellular matrix have long placed restrictions on the treatment of brain disorders. A prospective revolution in drug delivery for the treatment of AD is the use of nanomedicine. It depends on enhancing the way that medications are distributed pharmacokinetically throughout the central nervous system. Several types of nanoparticles (Nps) are available thanks to nanotechnology, and these Nps could target the brain and have a long half-life with few systemic side effects and motor problems. With the latest technological developments, scientists are working to develop unique approaches for the treatment of AD. To evaluate the prospective uses of medicinal plants, their components, and different nanomedicine techniques, it was determined that this literature study was necessary. To provide an overview of the various challenges and approaches related to using nanoparticles (NPs) to combat Alzheimer's disease (AD), this introductory review article was developed.

## 1 Introduction

### 1.1 Alzheimer disease (AD)

Alois Alzheimer originally provided a description of AD in 1906. A major public health issue, AD is one of the most complicated and debilitating neurodegenerative illnesses. The cerebral cortex exhibits significant shrinkage and cortical neuron loss in AD. The primary clinical sign of AD is short-term memory loss. As the illness worsens, other cognitive abilities are also compromised. It has recently gotten a lot of attention, particularly in areas involving new treatments. Clinical signs and symptoms of AD include increasing cognitive decline, impairment in daily living activities, and behavioral abnormalities that worsen with time, lowering quality of life.AD is the most prevalent neurodegenerative disorder in adulthood, occurring 1.5 times more frequently than stroke and as commonly as congestive heart failure. The worldwide prevalence of AD was 55 million in 2021, nearly ten million new cases every year, and expected to rise to 139 million in 2050 (WHO, 2022).

The cholinergic system plays an important role in learning and memory, and the severity of memory impairment in AD is primarily related to cholinergic dysfunction. Therefore, restoring cholinergic neurotransmission may aid in the treatment of assisting patients with AD. There are no specific therapeutic tools to prevent AD, therefore it is considered a top research priority. In modern therapeutic approaches to efficiently treat AD, various synthetic and natural molecules have been used to inhibit the cholinesterase enzyme which is responsible for the progression of AD. Studies have shown encouraging results using cholinesterase inhibitors to increase the concentration of acetylcholine in the brains of AD patients.

**Fig. 1:**
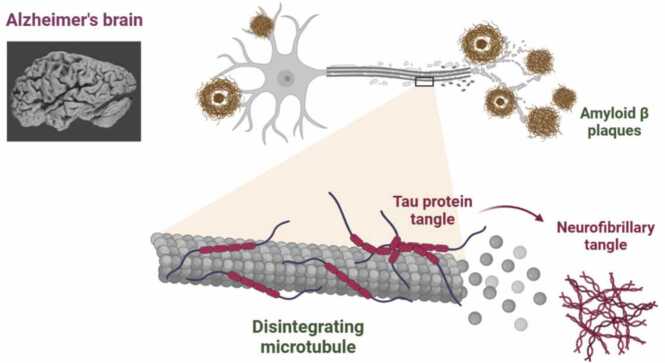
Neurodegeneration in AD with the Formation of amyloid-β plaques and Tau protein tangles.

Alzheimer's disease (AD) is characterized by the accumulation of amyloid-β (Aβ) plaques outside neurons and tau protein tangles inside neurons, leading to neuronal damage and cognitive decline. Aβ plaques disrupt cell communication, while tau tangles, also known as neurofibrillary tangles, cause the breakdown of microtubules, essential for nutrient transport, leading to further neuronal dysfunction. The exact cause of AD is complex and involves genetic, environmental, and lifestyle factors, with key mechanisms including aberrant Aβ production and clearance, tau protein dysfunction, and neuroinflammation. Together, these processes drive neurodegeneration and cognitive decline (Fig. 1).

Amyloid fibrils with crossed patterns as a misfolded state of the protein are a common feature in polypeptide chains [87]. The charge, hydrophobicity, and polarity of the amino acid sequence in the polypeptide, as well as environmental conditions, determine the propensity of amyloid fibrils. A high tendency for fibril formation is observed in synuclein, amyloid-beta peptides, polyglutamine, prion proteins, 2-microglobulin and glucagon inducing AD [50]. Protein aggregation and fibril formation, which also occur in apatogenic proteins were considered amyloidogenic templates for in vitro studies and were referred to as "myloid-like" [56],

Several studies have shown that the production of reactive oxygen species (ROS) is involved in the interaction of Aβ peptides with mitochondria and with resident proteins such as cyclophilin, alcohol dehydrogenase and Adenosine 5′-triphosphate (ATP) synthase [6], [7]. As this type of ROS- induced mitochondrial dysfunction precedes the accumulation of Aβ plaques in the brain, which is a hallmark of AD [184], [25], therapies that can protect mitochondria from ROS induced oxidative stress would be highly effective for early AD prevention.

Protein aggregation exhibits various structures from irregular amorphous deposits to regular amyloid fibrils [12]. The transient local folded or unfolded state in proteins can form soluble oligomers, which are the starting point for the stepwise fibrillation process. In this pathway, nucleation and growth phases induce various types of production from amorphous aggregates, dimers, globular forms, protofibrils to fibrils [130]. The size of amyloid fibrils differs between different proteins and peptides, ranging from a few nanometers to diameters > 30 nm, with lengths up to several micrometers [58].

The high stability of the amyloid fibril conformer hinders the therapeutic approach of this type of misfolded disease [36]. Preventing fibril aggregation creates the potential for disease-fighting amyloid protein. The structural protection of proteins during denaturing conditions arises from osmolytes, and chaperones [45]. Various groups of materials have been reported to inhibit fibril formation [102].

## 2 Methods

The relevant literature was searched using Science Direct, and Web of Science databases by covering the period from 2006 to 2024.The search was conducted using the following keywords: “Alzhaimer diseases”, “current therapies”, “phytocompounds”,“Nanoparticles”, and their equivalent terms. Editorial papers, commentary, and case reports were not included in this review.

### 2.1 Current therapies

**Fig. 2:**
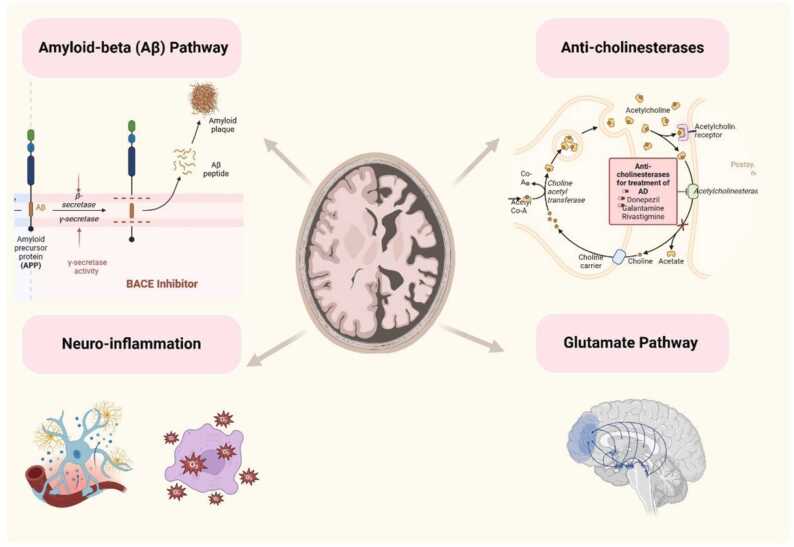
Alzheimer disease treatments targets.

Alzheimer’s disease treatments focus on several key targets (Fig. 2) such as reducing amyloid-beta (Aβ) plaques using monoclonal antibodies and BACE inhibitors, preventing tau protein aggregation, and modulating neuroinflammation through anti-inflammatory agents. Therapies also aim to enhance cholinergic and glutamate pathways to improve cognition and reduce neurotoxicity. Additionally, antioxidants and mitochondrial enhancers address oxidative stress and mitochondrial dysfunction. These strategies aim to slow disease progression, reduce symptoms, and improve cognitive functions.

There are now ChE inhibitors available to treat AD at all stages [24]. Many natural and synthetics compounds with the ability to thwart the cholinesterase enzyme have been published [81]. Within synapses and neuromuscular junctions, the enzyme catalyzes the transformation of acetylcholine into choline and acetic acid. Diminished levels of acetylcholine may be associated with the onset of Alzheimer's disease (AD). Presently, drugs that temporarily obstruct ChE enzymes have a fundamental effect on elevating ACh concentrations in the CNS.

**Fig. 3:**
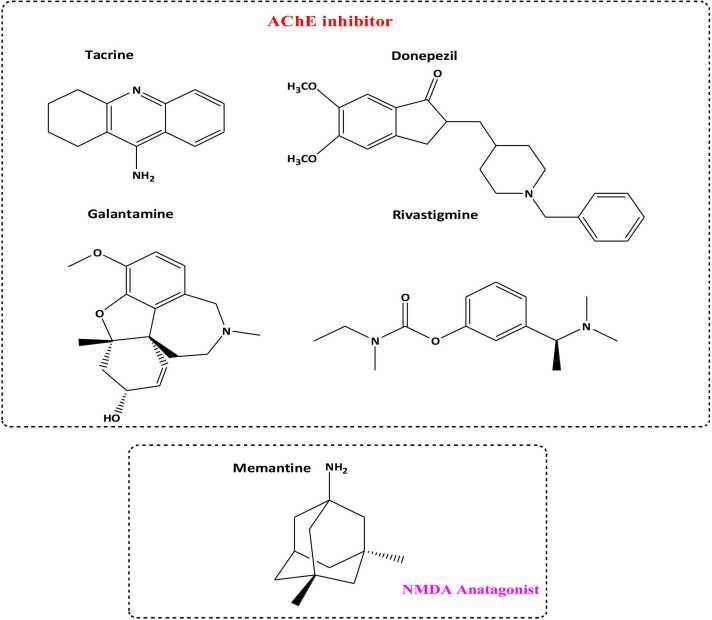
FDA-Approved drugs for AD as AChE inhibitors and NMDA antagonists.

These days, the FDA has only approved five medications to treat AD: memantine, galantamine, rivastigmine, and donepezil (Table 1, Fig. 3). The first is an antagonist of the N-methyl-D-aspartate receptor (NMDA), whereas the other four are AChE inhibitors [83]. Unfortunately, the current state of AD treatment choices reflects our poor understanding of the disease's pathological underpinnings, meaning that these medications solely treat its symptoms. Although they are unable to stop the disease's course, they can momentarily improve cognitive abilities by somewhat improving glutamatergic and cholinergic neurotransmission [63]. AchE inhibitors do, however, have certain drawbacks, including the possibility of gastrointestinal issues, variable absorption and bioavailability, and instability in the bloodstream [143].

**Table 1:** Pharmaceuticals sanctioned by the FDA for Alzheimer's disease.

**FDA** **Approved AD Drug**	**Class**	**Mechanism**	**Side effects**
Rivastimine	Cholinesterase inhibitor Severe AD	Prevent the breask down of butrylcholin and acetylcholine in brain	Nausea,loss of appetite, muscle weakness
Donepezil	Cholinesteraseinhibitor Moderate AD	Prevent the breask down of Acetylcholineinbrain	Nausea, diarrhea and vomiting
Memantine	N-methyl-D-aspartate antagonist Moderate to severe AD	Block the toxic effects associated with excess glutamate and regulate activation	Headache, dizziness and constibation
Galantamine	Cholinesteraseinhibitor Chemical massenger regulation Mild to moderate AD	Stimulate nicotinic receptor store lease more acetylcholine in the brain	Weightloss, vomiting and diarrhea
Combination drug Donepezil+Memantine	Severe AD	Combination effect	Nausea, loss of appetite,muscle weakness and weightloss

### 2.2 Existing obstacles

Since AD primarily affects the elderly population, polypharmacy, comorbidities, and nonadherence have a major impact on reducing the effectiveness of treatment regimens [123]. Currently available AD drugs have limited efficacy, including symptom relief, side effects, and are expensive [186]. The main cause of gastrointestinal side effects has been associated with stimulation of the peripheral cholinergic system [28]. In addition, these drugs have limited efficacy and prolong therapy [170]. The disadvantages mentioned can be attributed to the poor pharmacokinetic profile of these therapeutic components, including volatility, oxidative hydrolysis, low bioavailability, limited across blood-brain barrier and propensity for drug interactions [80]. Therefore, the use of nanotechnology to overcome this limitation has been reported in various studies.

Polypharmacy, comorbidities, and nonadherence have a significant impact on lowering the effectiveness of treatment regimens because AD mostly affects the older population [123]. Drugs for AD are already available, however they are expensive, only partially effective in relieving symptoms, and have negative effects [186]. The stimulation of the peripheral cholinergic system has been linked to the primary cause of gastrointestinal adverse effects [28]. These medications also prolong therapy and have low efficacy [170]. The poor pharmacokinetic profile of these medicinal components, which includes volatility, oxidative hydrolysis, low bioavailability, limited across the blood-brain barrier, and a susceptibility for drug interactions, is to blame for the drawbacks listed [80]. Hence, it has been reported that using nanotechnology to overcome this restriction.

### 2.3 Advantages of phytocompounds as potent therapeutics

Alzheimer's disease poses a major public health challenge in developed societies. Because flavonoids have been repeatedly reported to have anti-AD activities [5], [6] due to their ability to affect major mechanisms of AD, including mitochondrial dysfunction, neuroinflammation and oxidative stress, they have become increasingly important investigated for their anti-AD potential (Table 2). However, the medicinal properties of orally administered flavonoids are largely limited by their poor solubility, stability and bioavailability. In addition to using polymeric NPs in drug delivery. It has been reported that nanocomposites loaded with biologically active ingredients offer many advantages over their conventional formulations, including increased targeting, solubility, stability, bioavailability, permeability, and minimized side effects [103].

**Table 2:** Summary of evidence for therapeutic potential of phytochemicals against AD.

**PBC**	**Outcome**	**Reference**
**Curcumin**	Protection against Aβ-induced mitochondrial and synaptic toxicities in Human SH-SY5Y neuroblastoma cells	[138]
	Decreased secretion of TNF-α, prostaglandin E2 and NO; Inhibition of extracellular signal regulated kinase, p38, phosphorylation in Lipoteichoic acids-stimulated BV−2 malbinomiceoglial cells	[177]
	Reduction of oxidative damage, inflammation and amyloid pathology In APPSw(Tg2576) transgenic AD mice	[99]
	Reduction of soluble tau level in Human tau (hTau) transgenic mice	[104]
	Inhibition of Aβ aggregation, oxidative stress and inflammation in AD patients	[17]
	Decrease of cognitive deficits in animal models Reduction of BACE−1 mRNA Reduction of the formation of Aβ fifibrils Reduction of Aβ deposits and senile plaques in	[48], [71], [174]
	Tg2576 mice model Protect cells from Aβ-induced toxicity Reduction of Aβ-induced expression of cytokines and chemokines Reduction in oxidized proteins in Tg2576 mice model Increase of post-synaptic density−95 in vitro in the brain of Aβ-injected rats	[47], [51], [86], [99], [100], [124]
	Decrease of cognitive defificits in animal models Reduction of BACE−1 mRNA Reduction of the formation of Aβ fifibrils Reduction of Aβ deposits and senile plaques in Tg2576 mice model Protect cells from Aβ-induced toxicity Reduction of Aβ-induced expression of cytokines and chemokines Reduction in oxidized proteins in Tg2576 mice model Increase of post-synaptic density−95 in vitro in the brain of Aβ-injected rats	[48], [71], [174] [99] [100], [47] [51], [86], [124]
**Quercetin**	Attenuated Aβ1 −42-induced cytotoxicity, protein oxidation, lipid peroxidation and apoptosis at low doses; toxic effects at high doses In Cortical neuronal culture from Sprague-Dawley rat fetuses	[9]
	Preventing the toxic effects induced by H2O2 and, to a lesser extent, the Aβ-induced toxicityIn Culture of the hippocampal neurons from Sprague-Dawley rat fetuses	[53]
	Amelioration of mitochondrial dysfunction by decreased ROS production; Reduced scattered senile plaques; Rescued learning and memory deficitsInAPPswe/PS1dE9 transgenic AD mice	[167]
	Decreased β-amyloidosis, astrogliosis tauopathy,and malbinomiceogliosis; Improved performance on learning and spatial memory tasksIn Triple transgenic 3xTg-AD mice	Sabogal-Guáqueta et al., 2015 [132]
	No differences in MMSE and cognitive impairment rating scale scores in older subjects; Higher MMSE scores at week 24 in younger subjectsIn AD patients aged 65–84 years	Nishimura et al., 2017
**Resveratrol**	Remodels soluble oligomers and fifibrils form into nontoxic form of Aβ Reduction of production of Aβ peptides in vitro Decrease of ROS and lipid peroxides levels in animal models Decrease of cognitive deficits in animal models	[61], [62], [73], [107], [140], [149] Sharma et al., 2002 [91], [62], [94]
	Inhibition of Aβ-induced neuronal apoptosis in PC12 cells	[43]
	Amelioration of spatial learning memory impairment induced by Aβ1 −42In Hippocampal injection- induced AD rat model	Wang et al., 2017
	Amelioration of Aβ-induced learning and cognitive declineIn Kunming mice	[136]
	Reduced MMP9; modulated neuroinflammation; induced adaptive immunityIn Mild-moderate AD patients	[117]
	Differences in Aβ40 levels in the plasma and cerebrospinal fluid in comparison with placebo groupIn Patients with moderate AD	[161]
	Reduced glycated hemoglobin A1c levels; Preserved hippocampus volume; Improved hippocampal functional connectivityIn Patients with mild cognitive impairment (age - 50 −80 y)	[88]
	Enhancement of vasodilator responsiveness in cerebral vesselsIn Diabetic patients (49 −78 y) with sub-clinical cognitive impairment	[172]
**Genistein**	Inhibition of Aβ-induced production of COX−2, iNOS, IL−1β and TNF-αIn Primary culture of fetal cortical rat astrocytes	Valles et al., 2010
	Lowered Aβ levels in brain; Improved learning, recognition and implicit memory and odor discrimination In APPswe/PS1dE9 AD mouse model	Bonet-Costa et al., 2016
	Degradation of Aβ and hyperphosphorylation of tau proteins In Streptozotocin-induced AD rat model	[134]
**EGCG**	Inhibited aggregation of tau into toxic oligomersIn Neuronal model PC−12 cells	[171]
	Enhanced clearance of AD-relevant phosphorylated tau; Iincreased expression of autophagy adaptor proteins NDP52 and p62In Culture of primary cortical neurons isolated from E18 Sprague–Dawley rat embryos	[33]
	Reduced Aβ deposits and inhibited Aβ oligomerizationIn Transgenic TJ356 (DAF- 16:GFP)	[3]
	Prevention of amyloidogenesis and memory impairmentIn Lipopolysaccharide-induced neurodegenerative albino mice mice model	[95]
**Catechins**	Reduction in the translation of APP mRNA Increase α-secretase activity Reduction in the production of Aβ peptides in APP695 over-expressing neurons Reduction in β-secretase activity Reduction in the formation of Aβ fibrils by binding to the native unfolded Aβ Protect cells from Aβ-induced toxicity Reduction in Aβ-induced caspase activity in hippocampal neuronalcells Reduction in Aβ-inducedcytokines in human astrocytoma U373MGcells Reduction in Aβ-induced levels of lipid oxidation in hippocampal neuronalcells Decrease of cognitive defificits in animal models	[74], [140], [44] [37], [14] [23], [34], [62], [85], [96], [110], [125] [14], [23], [96]

Many natural compounds, especially phytochemicals, may have preventive and therapeutic potential for diseases [70], [115], [38]. However, most of these compounds have low levels of solubility, stability, bioavailability and target specificity in the body, which makes it unrealistic for these compounds to be present at their effective levels in the target tissues. This is particularly true for (−)-epigallocatechin gallate (EGCG) found in green tea, resveratrol found in grapes, curcumin found in turmeric and quercetin found in red onions, which are valuable for prevention and treatment of many diseases. Therefore, this represents an excellent opportunity for introducing NP technology to help resolve this issue (Table 2).

### 2.4 Advantages and disadvantages of current therapies and phytochemical compounds

Modern treatments come with several benefits, including the ability to control symptoms, alter the course of the underlying illness, demonstrate efficacy, and provide targeted care. However, it has a few drawbacks, such as side effects, accessibility and cost issues, treatment complexity, etc. Similarly, using phytocompounds in age-related brain disorders has several benefits: they come from natural sources; they have strong anti-inflammatory and antioxidant qualities; they may have neuroprotective effects; and they can be used as an adjuvant therapy. They can, however, restricted bioavailability, interact with other pharmaceuticals, thereby compromising their safety or efficacy, and there is no universal standardization or quality control. Therefore, it can be stated that recent developments in the treatment of age-related brain illnesses hold promise for better health care in the future due to the larger advantage when compared to the disadvantages surrounding present medicines and phytocompounds. Notwithstanding these drawbacks, new developments in phytochemical research and existing therapies hold out hope for better management of age-related brain problems down the road. To guarantee consistent clinical results, more research is necessary to completely comprehend the mechanisms of action of phytocompounds, determine the best dosages, and create standardized extracts.

### 2.5 Nanotechnology

Nanotechnology is a fast-expanding area of study because of its numerous applications in the medical sciences. To improve treatment efficacy and reduce hazardous side effects, a number of drug delivery systems have been created. Due of their capacity to distribute different medications to different parts of the body over an extended period of time, Nps have grown to be a significant study topic in the field of drug delivery. Nps are frequently employed for site-specific drug targeting and controlled drug release. The Nps can integrate, encapsulate, absorb, adsorb, or be linked to drugs. Numerous authors have used Nps as carriers to deliver a variety of medications. Nps offer an added benefit over larger microparticles in that they can be administered intravenously more easily.

**Fig. 4:**
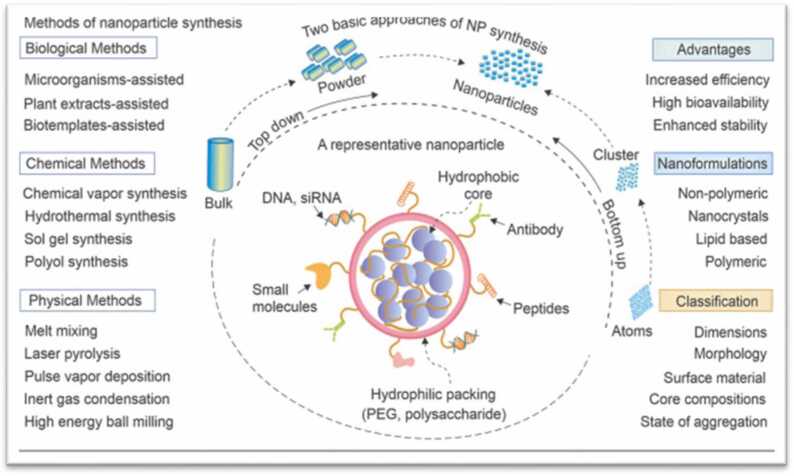
A brief synopsis of multipurpose NPs: Diagrammatic illustration of the two main NP synthesis procedures, which are carried out via physical, chemical, and biological synthesis techniques. For targeted medication delivery, a typical NP is adorned with a variety of ligands, antibodies, peptides, nucleic acids, etc. Various kinds of nanoformulations that are frequently employed to deliver drugs, along with some important classification criteria for these nanomaterials.

Nps have entirely better properties. There are several techniques for creating NPs in the liquid phase, and they typically call for the following four elements: the medium, the precursor which in the case of MNPs are metal salts, reducing agents, and stabilisers. Numerous physical and chemical processes, including electrochemistry, hydrothermal, laser ablation, lithography, microwave, and thermal decomposition, can be used to create Nps. All of these techniques, use dangerous chemicals, hazardous solvents, and expensive, occasionally unreliable, high pressure and temperature reactions that are bad for the environment and human health [118]. A concise overview of multifunctional nanoparticles illustrated in Fig. 4.

### 2.6 Advantages of using Nps for CNS-directed drug delivery: overcoming existing obstacles

Designing medicine delivery systems for AD is difficult because of blood-brain barriers. Drug dosage forms often have relatively low bioavailability and are unable to easily cross the blood–brain barrier; instead, they can only do so through active efflux or carrier–mediated transport [133], [16]. Studies conducted in vitro and in vivo support Nps' ability to cross the blood–brain barrier. They advocate for integrating nanotherapeutics into the brain so that Nps can be utilized for gene therapy, brain-directed therapy, and diagnostic purposes in addition to treatment [18], [20], [178].

Nanotechnology, a broad topic, can be a hotspot for the discovery of new treatment techniques, such as drug delivery.Currently, NPs have a multitude of applications within the field of medicine, including both diagnostics and drug delivery systems. Due to their diminutive size, adaptable morphology, and customizable surface features, NPs present themselves as exceptional candidates for circumventing any obstacles that impede medication delivery systems in the brain [101]. NPs are suitable for improving the brain-to-blood ratio of already approved drugs by transporting larger amounts of drug across the blood-brain barrier and interacting with amyloid in the brain due to their high loading capacity [42], improving targeted drug delivery across the blood-brain barrier (Rocha, 2013), boosting the potency of the already available approved drugs, and offering a multifunctional platform that can help with theranostic application [109].

### 2.7 Various techniques have been utilized to enhance the efficacy of AD pharmaceuticals for more effective treatment

(1) Hydrophilic polymer-based NPs act as a sustained release parenteral medication system and confer stealth properties by prolonging the blood retention time [169].
(2) Lipophilic NPs, like liposomes, improve the blood-brain barrier crossing of hydrophilic medicines [153].
(3) NPs that are receptor-mediated transcytosis-targeted with surface ligands [162].
(4) By facilitating direct uptake across the blood-brain barrier through endocytosis, mucoadhesive polymeric NPs have been employed to improve nasal medication delivery [112].

Overall, Nps have a number of promising benefits that can be used in the medical field to produce advanced new dosage forms, including high drug loading capacity, which lowers the likelihood of toxic or chemical interactions, high surface area to volume ratio, ease of handling for parenteral administration due to Nps' particle size and surface characteristics, ability to be used for active drug targeting strategies, objectives of sustained and controlled drug release, and more [55].

NPs use several different methods to transfer drugs across the blood-brain barrier: (a) transporting drugs through tight junctions; (b) transporting drugs via cellular transport proteins;(c) NP transport by receptor-mediated transcytosis; (d) NP transport via diffusion uniquely adopted by gold nanoparticles; and (e) NP transport via adsorption-mediated transcytosis for cationic nanoparticles and liposomes.

### 2.8 Ideal properties of Nps for drug delivery in the brain

(1) Nps must be biocompatible, biodegradable and non-toxic [108].

(2) The cleaning rate increases with increasing particle size above 100 nm, affecting both biodistribution and bioavailability. The NP size is preferably less than 100 nm [66].

(3)When specific forms of Nps are regarded as harmful based on physical rather than chemical surface features, physical stability and avoidance of aggregation in blood are necessary.

(4) Extended circulation time, with PEGylated Nps being the only ones to exhibit reduced absorption and a longer time [127].

(5) Tailored delivery to the brain without active reflux or carrier-mediated routes to boost medication bioavailability and effectiveness and lower needed therapeutic dose and consequent efficacy [52].

(6) Gene-directed non-invasive delivery to the brain, such as PEGylated immune liposomes by receptor-mediated transcytosis, which transports their contents to brain tissue without compromising the blood brain barrier [78].

(7) Cost-effectiveness studies ought to assess the possible application of Nps in medicine. The decreasing reimbursement rates will reduce the number of people who can use this medication (Parikh, 2016). (Table 3)

**Table 3:** Advantage and limitation of different classes of Nps used in AD therapy.

	Inorganic Nps	Organic lipid based Nps	Organic polymeric Nps
Advantages	Increased uptake due to ionic interaction with BBB	Ease of ligand conjugation to improve blood circulation Use for hydrophilic and lipophilic cargo	Biodegradable Adjustable surface modification Use for hydrophilic and lipophilic cargo
limitations	Potential cytotoxicity caused by metal accumulation	Potential cytotoxicity caused by non-specific uptake	Self-aggregation may impact brain delivery

### 2.9 Biogenic NPs as a future promising therapeutic agent in AD

There are numerous disadvantages to producing Nps using conventional methods [92], [30]. Therefore, the creation of non-toxic and eco- friendly processes to synthesis these beneficial Nps is urgently needed.

Recently, effective alternatives to the production of Nps have been discovered in medicinal plants. This method, known as the biogenic approach, uses medicinal plants to produce valuable chemicals that can be of great concern in the treatment of AD and cancer [128], [163], [185]. These organisms are abundant in nature and have the potential to convert metals into NPs using a straightforward synthesis while also maintaining the stability of the NPs produced. Nps can be produced using this ecologically friendly method without using dangerous chemicals or under difficult reaction circumstances.

Nevertheless, green synthesis is the preferred method for producing Nps due to its accessibility, environmental friendliness, biocompatibility, ease of usage, and short synthesis time [65], [151]. The environmentally friendly Nps that are developed offer an alternative to AD medicines as a delivery technology for targeted and secure drug distribution.

Moreover, the primary benefits of this biogenic synthesis include its speed, efficiency, safety, and ability to produce Nps with distinct morphologies and sizes [106], [41]. Several plant components can serve as capping and reducing agents in biogenic processes, decreasing the need for toxic reducing agents such sodium lauryl sulfate, sodium borohydride, and hydrazine, which may alter how other reducing agents can be employed [82].

### 2.10 The mechanistic principle of NPs therapy

**Fig. 5:**
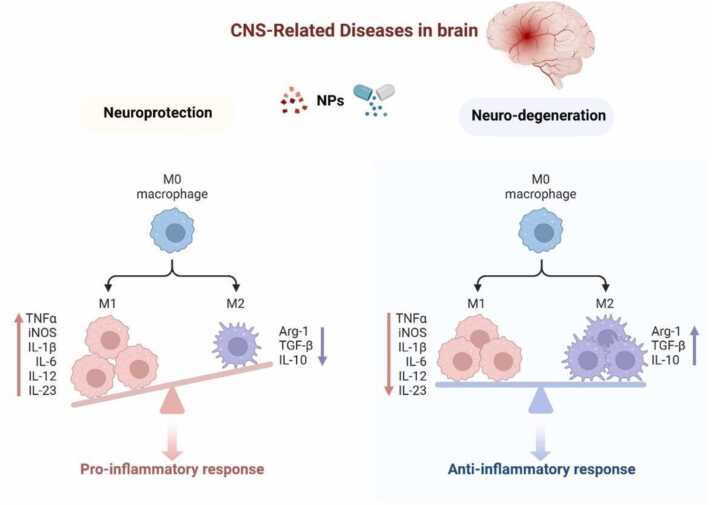
The potential therapeutic impact of NPs on AD [183].

Nanoparticles (NPs) in Alzheimer’s disease (AD) therapy encapsulate therapeutic agents targeting amyloid-β and tau proteins to prevent their accumulation and reduce plaque formation. NPs also act as antioxidants, alleviating oxidative stress in neurons and modulating immune responses to decrease neuroinflammation. Mechanistically, NPs promote amyloid-β clearance by activating microglial phagocytosis through the PI3K/Akt pathway, enhancing cell survival and amyloid breakdown. They also reduce oxidative stress by modulating the Nrf2/Keap1 pathway, which regulates antioxidant protein expression. Furthermore, NPs influence the NF-κB pathway to lower inflammatory cytokine production and mitigate neuroinflammation. Additional pathways include RAGE and LRP1 for amyloid management, GSK-3β and CDK5 for tau phosphorylation inhibition, and PP2A for tau dephosphorylation. TLR4, NF-κB, and TREM2 are also targeted to control inflammation and support neuroprotection. This multi-pronged approach demonstrates NPs as both delivery agents and modulators of critical AD pathways, providing a comprehensive therapeutic strategy against neurodegeneration (Fig. 5).The unidentified pathophysiology and rising older population point to a growing need for the creation of AD-specific treatment compounds. In an effort to identify the fundamental cause or limiting factor of AD, numerous researchers from around the world have created NPs that target various molecular pathways [120]. The pathophysiology of AD can be targeted with NP at four key and basic sites. A neurotransmitter modulator, a metal stress reliever, an anti-amyloid beta immunotherapy, an inflammation inhibitor, and a neurotransmitter modulator (Fig. 5).

## 3 Anti-inflammatory agent

Anti-inflammatory Agent: CoQ10-based formulations using trimethylated chitosan (TMC) and PLGA nanoparticles have shown improved brain accumulation, biocompatibility, and reduction in memory impairment. These formulations reduce oxidative stress and amyloid-beta (Aβ) concentrations [49], demonstrating potential in mitigating mitochondrial damage and neuroinflammation in AD.

## 4 Neurotransmitter modulator

Nanoparticles (NPs) carrying drugs like rivastigmine are designed to cross the blood-brain barrier, modulating acetylcholine (Ach) levels to correct neurotransmitter imbalances, improve memory, and alleviate cognitive deficits in animal models [72].

## 5 Immunotherapy directed at beta amyloid

chitosan-based NPs enhanced the transport of Aβ subfragments across the blood-brain barrier, stimulating immune responses and targeting amyloid deposits. Studies show these formulations can effectively trigger immunogenicity and improve amyloid clearancey [7].

## 6 Metal chelator

Metal ions (e.g., Cu2 +, Zn2 +) contribute to Aβ aggregation in AD. NPs loaded with metal chelators, such as ruthenium-based and polystyrene compounds, prevent metal-induced Aβ fibrillation and scavenge reactive oxygen species (ROS), offering neuroprotection and reducing cytotoxicity. These strategies emphasize the importance of optimizing NP properties (size, charge, and modification) for effective metal chelation.

## 7 Promising nanomedicine trials

### 7.1 Nanomedicine for delivering FDA-approved Alzheimer’s drugs

An overview of the most recent uses of NPs as carriers to deliver FDA-approved Alzheimer's medications is given in Table 4. The table highlights the many NP types, their loaded cargo, their material composition, the administration route, and the primary results noted.

**Table 4:** Recent applications of NPs for delivering FDA-approved Alzheimer’s drugs.

**NPs Type**	**Results**	**Ref.**
PEG / Donepezil Liposomes	liposome-based sustained release of donepezil and improved bioavailability in the brain and plasma.	[8]
Chitosan / Donepezil Polymeric	NPs increased the drug's concentration in the target tissue by improving its pharmacokinetic characteristics and bioavailability.	[22]
PLGA / Donepezil Polymeric	Drug concentration in the target tissue rose as a result of NPs' considerable increase in drug transport to the brain.	[21]
PLGA-PEG / Donepezil Polymeric	NPs were able to effectively deliver donepezil across the BBB and release it in a controlled manner.	[19]
Dynasan / Donepezil Polymeric	NPs demonstrated enhanced permeability, more cellular absorption, and a prolonged release of the medication.	[160]
cholesterol, and PG /Galantamine Polymeric	Liposomes have the potential to improve AChE inhibition and transport galantamine via the nose-to-brain pathway with enhanced pharmacokinetic behavior.	[97]
PLGA / Galantamine Polymeric	NPs are expected to increase therapeutic effectiveness and decrease negative effects because they gave the medicine a longer-lasting release than galantamine solution.	[46]
Glyceryl Behenate / Galantamine SLNs	SLNs offered a regulated release, altered the drug's temporal course in vivo, and improved its bioavailability.	[111]
Chitosan // Galantamine Polymeric	NPs improved the pharmacodynamic behavior of the medication. The rats who received the NPs recovered considerably from induced amnesia and showed increased AChE levels.	[157]
MPEG-PCL / Rivastigmine Polymeric	NPs were able to enhance memory impairment by delaying the release of the drug and increasing rivastigmine's in vivo brain uptake clearance.	[114]
Chitosan / Rivastigmine Polymeric	In comparison to rivastigmine solution, NPs provide a controlled and prolonged release of the medication with better brain targeting efficiency.	[42]
Soya lecithin /Rivastigmine Liposomes	NPs were able to enhance memory impairment by delaying the release of the drug and increasing rivastigmine's in vivo brain uptake clearance.	[142]
Lecithin, and Tween® 80/ Rivastigmine Liposomes	Liposomes increased rivastigmine's bioavailability and delayed its release. The drug levels were almost quadrupled in both the brain and bloodstream.	[40]
Phosphatidylcholine, Dihexadecyl phosphate, cholesterol, and glycerol /Rivastigmine Liposomes	The medication was released via liposomes in a controlled and prolonged manner. Additionally, the application of nanocarriers led to a marked improvement in cognitive impairment and an increase in AChE activity.	[72]
Cholesterol, Lecithin, oleic acid, PG, and PEG / Rivastigmine Liposomes	Following topical administration, liposomes preserved plasma levels within the therapeutic window and improved rivastigmine penetration through the skin.	[146]
Glyceryl Behenate / Rivastigmine SLNs	SLNs increased the drug's nasal penetration both in vitro and ex vivo. Its safety for intranasal delivery was demonstrated by the nasal mucosa's preservation.	[150]
Glyceryl monostearate / Rivastigmine SLNs	In vivo, SLNs enhanced the drug's pharmacokinetic profile, bioavailability, and concentration in the brain and plasma.	[10]
Silica /Rivastigmine Organic NPs	NPs enhanced the drug's pharmacokinetics parameters and enabled a prolonged release in vitro.	[13]
PLGA / Memantine Polymeric	Because NPs extended the duration of the drug's release, oral administration is used less frequently. In vivo, memantine-loaded NPs decreased inflammation and β-amyloid brain plaques linked to AD while also enhancing learning.	[147]
PAMAM / Memantine Dendrimers	The drug's pharmacokinetic characteristics were enhanced using dendrimers. In vivo, the DDS demonstrated a notable improvement in memory and behavioral responses.	[57]

### 7.2 Promising trials using nanophytocompounds

The delivery of neuroprotective phytochemicals in AD may be improved with the aid of nanotechnology. However, because of their limited solubility, low bioavailability, and poor blood brain barrier (BBB) penetration, these natural compounds frequently have poor therapeutic efficiency despite their amazing neuroprotective qualities. Personalized medication delivery methods, made possible by nanotechnology, are essential for resolving these issues. The development of personalized drug delivery systems could be facilitated by the use of nanotechnology in addressing these obstacles.

Nanophytocompounds show promise for Alzheimer’s disease treatment by efficiently crossing the BBB and targeting multiple AD pathways. Derived from plant compounds like curcumin, quercetin, and bacoside, these nanoparticles have neuroprotective, anti-inflammatory, and antioxidant effects. Functionalizing these compounds enhances their stability, bioavailability, and efficacy, allowing targeted and sustained therapeutic delivery (Table 5).

**Table 5:** Nanophytocompounds providing neuroprptective effect in AD therapy.

**Nanophyto compound**	**Mechanism**	**Ref**
BacosideNPs	Cross blood brain barrier in vivo	[76]
Lectin-NPs	Bind to acetylglucosamine on the nasal epithelial membrane Improve memory in ADrat	[179]
HuperzineNPs	Crossbloodbrainbarrier	[110]
GinsenosideNPs	Crossbloodbrainbarrier	[1]
CholineNPs	Exhibit high gene expression and distribution High permeability across capillary cells	Lietal., (2011)
QuercetinNPs	Increase efficacy of system iccirculation	[131]
QuercetinNPs	InhibitAB42fibrilationReduce AB42-induced toxicity Enhane memory in ADmice	[156]
	BlockNFTsandAPsformationRegulatemicroglialandastrocyteactivities Improveneurona lfunction Minimize the degenerative changes of AD affected hippo campus Preserve myelinsheath Enhance regenerative changes	[141]
CoumarinNPs	Increase Aβ-degrading enzymes Reduce amyloidplaques Improves patiall earning and memory Protecting synapses Reduce tauprotein phosphorylationin APP/PS1transgenic mice	[182]
EGCGstabilizedseleniumnanoparticles	AβpeptidesAβaggregationandcytotoxicity/protective effect	[179]
CurcuminNPs	Improve memory in Tg2576 ADmice	[32]
CurcuminecapsulatedPLGA NPs	Induce neuralstem cells poliferation Induce neural differentiation invitroandin vivo inadultrats	[159]
Curcumin and selenium Nanospheres	Decreases the amyloid-βinTransgenicmice	[69]
VitaminD-bindingproteinNPs	Reduce Aβaggregation and accumulation in Aβ-overexpressing mice	[75]

**Fig. 6:**
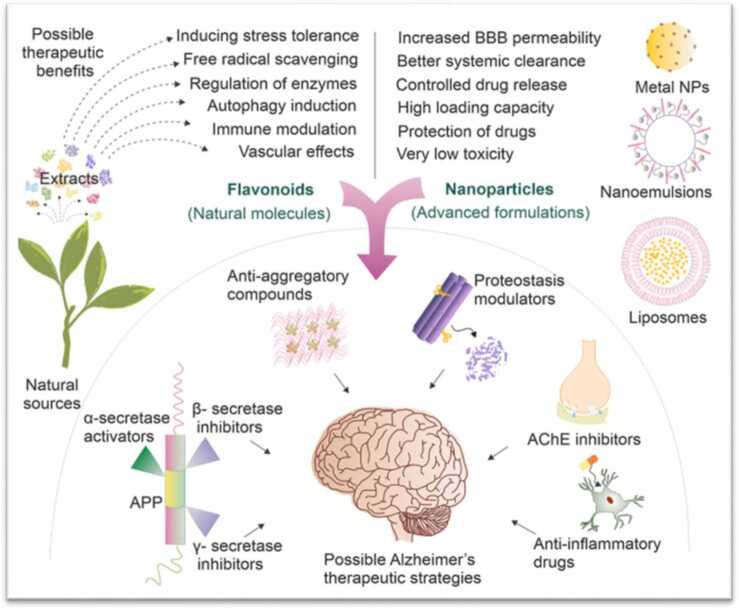
Therapeutical activity of phytocompounds nanoformulations in AD.

Plant-based phytochemicals functionalized on NPs, such as EGCG-coated selenium nanoparticles (SeNPs), show potential in inhibiting Aβ fibrillation and degrading amyloid plaques. Other compounds like curcumin and its derivatives are also explored for their affinity to amyloid proteins, highlighting the potential of natural compounds in AD treatment [181] (Fig. 6).

### 7.3 Transport mechanisms for Nanophytocompoundsover the BBB

**Fig. 7:**
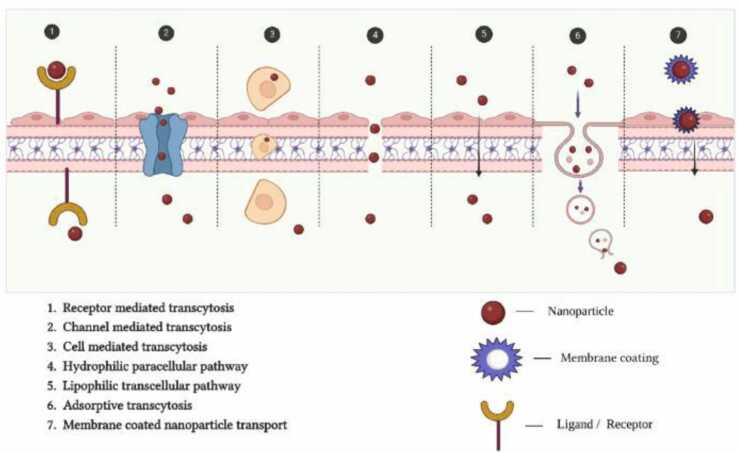
Schematic representation of various transport mechanism involved in the delivery of nanoparticles into the brain to achieve targeted delivery of drugs.Figure adapted from [79].

Encapsulating phytocompounds in NPs is a way to overcome several physicochemical limitations of drugs. ex, phytochemicals may be encapsulated within bio-degradable, bio-compatible NPs to increase water solubility, bioavailability, and stability [122] (Fig. 7).

5.3.1. Passive BBB transport

5.3.1.1. Transmembrane diffusion

5.3.2. Active BBB transport

5.3.3. Carrier-mediated transport

5.3.4. Receptor-mediated transcytosis

5.3.4.1. Transferrin receptor mediated brain delivery

5.3.4.2. Insulin receptor mediated brain delivery

5.3.4.3. Low-density lipoprotein receptor and low-density lipoprotein receptor-related proteins 1 and 2 mediated brain delivery

5.3.4.4. Nicotinic acetylcholine receptor mediated brain delivery

5.3.5. Adsorptive-mediated transcytosis

5.3.5.1. Peptide carriers as brain delivery shuttles

5.3.5.2. Macromolecules as brain delivery shuttles

### 7.4 Biogenic synthesis mechanism

There are several methods for determining the nature of interactions between plant extract biomolecules and MNPs [64], [4]. These biological sources are made up of biomolecules like proteins, enzymes, and polysaccharides that catalyze the conversion of metal precursors to NPs [35]. They can function as surfactant-type molecules, lower surface tension and interfacial energy, influence how molecules are oriented on the NP surface, and enhance stability because they are big, amphiphilic molecules. The use of compounds that can lower this energy is necessary because NPs with high surface and/or interfacial energies are unstable and have a propensity to assemble quickly [152].

It is known that some biomolecules can also function as surfactant-type molecules because of their amphiphilic nature, which influences the surface tension and surface orientation of the molecules. As a result, these molecules can also function as capping agents and reduce surface energy [11].

Numerous factors, including targeting properties, NPs size, hydrophobicity or hydrophilicity, physical and chemical stability, permeability, surface charge, biocompatibility, biodegradability, cytotoxicity, end product antigenicity, and drug release profile, affect the choice of material method for the production of Nps [39].

### 7.5 Recent applications of Nps in the management of AD

Anti-amyloid therapy, oxidative stress reduction, and blood-brain barrier crossing for brain-directed activities are the core objectives of AD therapy [119]. Bolus injections of the medication Nps into the brain have various benefits, including the achievement of high brain concentrations without affecting or decreasing blood-brain barrier function, an increase in cerebral cortex vascularity, and a decrease in beta amyloid deposits [126].

### 7.6 Metal nanoparticles (MNps) in AD therapy

The utilization of MNps is growing as a result of their unique physical, chemical, optical, and mechanical characteristics. In recent years, numerous researchers have been drawn to the biosynthesis of MNps due to its many benefits over traditional synthesis as a result of advancements in medicinal plant research and nanotechnology (fast, facile, safer, energy efficient, more economical, biocompatible and one-pot process). The finest sources of varied phytochemicals for the manufacture of biogenic Nps have been found to be medicinally bioactive plants.

The eco-friendly MNPs of silver, gold, iron, nickel, platinum, zinc, calcium and palladium have been synthesized by various biogenic approaches, such as using plants, bacteria, and fungi. Among all these biogenic MNps are of considerable interest due to their diverse applications, especially in clinical medicine, biomedical fields [145], [168] (Table 6).

**Table 6:** Therapeutic efficacy of different MNps in AD therapy.

**Nps**	**Effect**	**Reference**
biogenic platinum NPs produced by Bacopa monnieri	Prevent free radical production	[121]
Zn-PLGANPs	Affect proinflammatory release cytokinesIL−6andIL−18 Reduce plaque size in Wild-typeandAPP23mice	[165]
Biogenic AgNPs	Antioxidant activity and anti-ACHE in adults prague dawleyalbino male rats	[176]
AgNPs	Glutathione gene expression inmicehippocampus	[137]
AgNPs	Induction and progression of AD	Huang et al.2014
Selenium-containing clioquinol derivatives	Cu(II)-induced Aβ oxidation and aggregation Protective effect in Neuroblastoma cell line	[167]
Sialic acid-modified SePC12	Crossblood-brain barrier Reduce Aβ aggregation in neuronalcellline	[175]
PEG chain to Aβ1–42 peptidemonoclonal antibody	Enhance Aβ1–42 elimination through the “sink effect”in AD-like transgenic mice	[27]
BiphenylethersconjugatedCdSe/ZnS core	Aβ fibrildisruption	[59]
ZnO	Decrease SOD and GSH-Pxinadultalbino male brain	[164]
Biogenic lead oxide Nps	Inhibit ChE enzymes	[84]
Pdhydride NPs	Reduce oxidative stress	[180]
AuNPs	Inhibit of PC12 cells	[68]
AuNPs	Inhibit of Aβ42 aggregation	[67]
Selenium/ Curcumin-loaded nanospheres	Decrease amyloid-β plaques Curcumin binds with β amyloid and iron in plaques	[69]
AgNPs Citrate cap	Reduce neurotoxicity	[54]
Cerium-containing NPs (CeONPs)	Increase blood-brain barrier absorption and lack of unintended accumulation in other biological locations	[144], [166]
	CAT and SOD`mimetic activity	[31], [154]
triphenylphosphonium-conjugated CeONPs	Suppress neuronal death in AD mouse model by attenuate reactive gliosis	[93]
Gold AuNPs	AuNPs are attached to a collection of Aβ fibrils and destroying it, also lessening the propensity of the protein to combine.	[89], [15]
	small hydrodynamic diameter, small core size, positive zeta potential, hydrophobicity, and good colloid stability	[116]
	AuNPs block Aβ fibrillation Develop spherical oligomers and fragmented fibrils Strongly linked to the fibrillar structure. Co-incubated with Aβ than were amine-conjugated AuNPs. Control and prevent Aβ fibrillation,	[98]
biosynthesized AuNPs using Paeoniamountan root extract	Reduce neuroinflammation and improve motor control in mice affected by AD	[173]
Terminalia arjuna produces biogenic AuNPs	Decrease Aβ fibrillation processes, inhibit ChE enzymes -destroyed mature fibrils.	Sugarnthy et al. 2018
Trehalose-functionalized AuNPs	Prevent protein aggregation Disintegration of mature fibrils Inhibit ache Reduce Ab fibrillation processes Inhibit protein aggregation	[105]
AuNPs	- inhibit che disrupting mature fibrils,	[129]
Biosynthesized of AgNPs from the *Mucuna pruriens* L. seed extract	Reduce symptoms catalepsy in rats	[148]
AgNPs biosynthesized useing*Nepenthes Khasiana* extract	Avoid impaired object recognition performance Inhibit ChE enzymes	[29]
Biosynthesized AgNPs from *Malephora lutea* and Lampranthuscoccineus extracts	Reduce oxidative stress	[60] [176]
AgNps	Prevent the formation of amyloid fibrils in reduced lactalbumin.	[158]
Sialic acid (SA) on the surface of SeNps(SA@SeNps)	lysing Aβ fibrils and inhibiting Aβ aggregation, shield PC12 cells from the toxicity caused by Aβ. possesses high blood brain barrier permeability, outstanding anti-amyloid, and antioxidant capabilities	[175]
	Se-containing clioquinol derivatives in Cu(II)-induced A-oxidation and discovered that these compounds had positive effects on hydrogen peroxide scavenging activity, intracellular ROS production, and induced Aβ-aggregation in SH-SY5Y neuroblastoma mobile channel.	[167]
SeNPs	Prevent Aβ aggregation	
Tet−1-EGCG@SeNps	Prevent Aβ aggregation and fibrillation and shield PC12 cells from oxidative damage	[181]
ZnONPs	Accumulate in synaptic vesicles as part of glutamatergic neurons to support brain health	[77]
ZnONPs	Increase CNS neural excitability by releasing glutamate	[113]
ZnNPs	Transport to the brain via the blood brain barrier to prevent neurotoxicity	[139]
	Stabilize the neural proteins and make them less susceptible to oxidation	[90]
Biogenic ZnONPs	Potent antioxidants and strong protectors against pro- oxidant-induced carcinogenicity and tumorigenicity	[2]
	act as a cofactor of SOD to promote the formation of metallothionein to induce scavenging	[77]
	Modulating the NO radicals in the brain.	[26]
Iron NPs	Strong inhibitor of tau aggregation	[155]
Iron NP sprepared by Convolvulus pluricaulisaqueous extract	Improve memory and learning in normal rats and rats with scopolamine-induced amnesia.	[135]

## 8 Limitations

Most of the current results for these innovative drug delivery systems are either in vitro or in the mouse model and are only preliminary. When using these NPs in people for clinical purposes, there could be numerous difficulties. Any alteration in the functional activities of a tissue or cell that the NPs come into contact with while moving toward their destination is a little-studied topic. Furthermore, nothing is known about whether and how NPs alter the electrical impulse conduction of the neurons they target. The possibility that nanoparticles themselves are cytotoxic and that their administration could result in neurotoxicity is also a crucial factor to take into account. Furthermore, because these nanomaterials disrupt the integrity of the blood-brain barrier, they may open a channel for the entry of pathogens or harmful chemicals into the brain in addition to therapeutic medications. Moreover, NPs have the ability to disrupt regular cellular metabolism, which raises ROS and modifies gene expression. Even though there are still many unanswered questions, a lot of research is being done, and every advancement in nanotechnologies for medication delivery gets around the current problems. Drug delivery by nanoparticles is becoming more and more significant. A hopeful era in the treatment of AD may be unlocked by new targets such as mutant genes, DNA synthesis, hypoxia, neuroproteins, and neuropilin-1. Additionally, innovative therapeutics such as virus-, protein-, and nucleic acid-based NPs with improved BBB penetration could be employed.

There are still a lot of questions and gaps in the field of NPs application in biology. Even though the research of NPs for biomedical applications has advanced significantly over the past ten years, there are still numerous obstacles to be addressed before these materials are routinely used in clinical settings. More work is needed to develop innovative nanomedicine from the lab to the patient's bedside. Two main concerns with NPs are their toxicity and safety. While many NPs show promise in terms of their effectiveness for drug delivery, imaging, and other biomedical applications, it can be challenging to assess their safety using traditional evaluation techniques due to their peculiar properties, which can also lead to unpredictable toxicity and unanticipated effects on biological systems. Examining the safety profiles of nanomedicine is difficult because to various factors, such as the way in which it is administered, the intricate cellular makeup of the brain, and the modifications made to nanomedicine. It is imperative to thoroughly assess the long-term safety of these chemicals, both in vitro and in vivo, in order to reduce the possibility of unanticipated consequences.

Another difficulty is the requirement for standardization in the synthesis and characterization of NPs. For example, because there are currently no widely established criteria for the characterization and quality control of NPs, it may be difficult to compare the findings of different research or to accurately assess the performance of different types of NPs.

Lastly, the lack of understanding regarding the physiological and pathological pathways underlying AD further impedes the development of therapeutics based on NPs. As research into the underlying molecular causes of AD progresses, greater emphasis will likely be placed on NPs-based therapeutics.

## 9 Future prospects

NPs have the ability to attach to diverse molecules and transport them to distinct locations throughout the brain. In order to treat AD, these NPs can also bind several therapies with different purposes, possibly producing synergistic effects. Moreover, NPs allow for the simultaneous administration of therapy when paired with both therapeutic and diagnostic substances. Antibody fragments and NPs work together to create a fantastic theranostic system. Despite the availability of several NPs and antibodies, powerful combinations to create more successful nanomedicine-based immunotherapy for AD have not yet been developed. It is necessary to identify a novel immunotherapeutic target or NPs combination that ensures BBB penetration. Tau protein immunotherapies based on nanomedicines are currently scarce. To validate the development of tau protein immunotherapy employing nanomedicine, more studies in animal models are required. Furthermore, to ascertain the safety of medication formulations, a comprehensive in vivo evaluation of the dose, route of administration, and dosage form is required. To determine the toxicological profile, any alteration or change in the medication's composition requires additional testing. All things considered, using NPs seems to be a promising method for treating AD. Nevertheless, more thorough research is required to provide a reliable and effective nanomedicine against AD.

## 10 Conclusions, and viewpoint

The development of therapeutic drug efficacy in the treatment of AD is the goal of NPs-targeted brain drug delivery, which attempts to enhance clinical outcomes. This method of successful symptomatic therapy has received a lot of attention and is still being worked on in order to slow the progression of AD and lessen the known harmful side effects of therapeutic treatment. A number of NPs have been patented for the treatment of AD; some are still in the experimental stage, while others have been reported to be cytotoxic. There is currently no proof of the efficacy and safety margins of utilizing Nps in AD patients, despite the fact that research studies are still being conducted and all of these studies indicate promise for the prevention of AD.

Nanotechnology can be used to more effectively deliver medications to the target region, increasing the potential of bioactive chemicals. It is known that a number of NPs have a sizable capacity to scavenge radicals in the brain. While the majority of the NPs created to date have been through chemical processes, which are linked to these adverse effects, green chemistry-based NPs may be safer and more efficient. Because the field of green nanofabrication is relatively young, further work in biogenic fabrication may pave the way for novel approaches to the treatment of AD.

Even though these studies have shown encouraging findings, more investigation is necessary to properly understand any potential security issues with this particular type of structured NPs. Because of this, caution must be used when thinking about using NPs in clinical applications, and toxicity tests must be performed.

## CRediT authorship contribution statement

**Hamdy Rania:** Writing – review & editing. **El-Keblawy Ali:** Writing – review & editing. **Mahdy Ahmed:** Writing – review & editing. **Abou Baker Doha:** Writing – review & editing, Writing – original draft, Project administration.

## Declaration of Competing Interest

The authors declare that they have no known competing financial interests or personal relationships that could have appeared to influence the work reported in this paper.

## Data Availability

Available.
